# Prevalence and severity of periodontitis among adults in Côte d’Ivoire according to the new EFP/AAP periodontal disease classification

**DOI:** 10.34172/japid.2021.018

**Published:** 2021-11-28

**Authors:** Nadin Thérèse Koffi-Coulibaly, Zocko Ange Désiré Pockpa, Gnaba Samson Mobio

**Affiliations:** ^1^Department of Periodontology, Dental College, Felix Houphouet Boigny University, Cote d’Ivoire

**Keywords:** Periodontitis, prevalence, extent, severity, risk indicators, Côte d’Ivoire

## Abstract

**Background:**

To describe the prevalence and severity of periodontitis in patients attending the Periodontics Service of the Cocody University Hospital at Abidjan.

**Methods:**

This retrospective observational study reviewed records of patients aged 20-80 years who attended the Periodontics Service between January 2014 and December 2018. Periodontitis was diagnosed, according to the 2018 EFP/AAP new classification of Periodontal and Peri-Implant Diseases and Conditions. Chi-square test, 1-factor Anova test, and logistic regression were performed for analysis.

**Results:**

A total number of 596 patients were included. The mean age was 44.94 ± 14.34 years and 59.20% of were males. 2 (0.40%) patients were classified as Stage I, 221 (37.08%) as Stage II, and 373 (62.58%) as Stage III/V; the extent of periodontitis was generalized in 39.77% of patients. PD ≥ 6 mm, missing teeth ≥ 5 and mobile teeth were present in 47.15%, 26.35% and 25.50% of the sample, respectively.Severity of periodontitis were associated with age (p < 0.001), socio-economic status (p=0.001), diabetes (p < 0.001), missing teeth (p < 0.001) and smoking (p=0.009). Age (OR= 1.59, 95% CI: 1.11-2.26) and missing teeth (OR= 2.31, 95% CI: 1.08-4.89) were identified as independent risks indicators.

**Conclusion:**

The prevalence and severity of periodontitis were high. Risks indicators identified may allow early detection and management of groups at high risk in Côte d’Ivoire.

## Introduction

Periodontitis is a multifactorial inflammatory disease affecting the periodontal tissues.^1^ It results from a deficient host response following dysbiosis of the bacterial biofilm and is most commonly detected in adults but can also occur in children and adolescents.^2,3^ Periodontitis is characterized by progressive and irreversible destruction of supporting tissues of the tooth, manifest through clinical attachment loss (CAL) and radiographically assessed by alveolar bone loss, the presence of periodontal pocketing, and gingival bleeding.^4^ If untreated or inadequately treated, it may lead to dental mobility and tooth loss.^4,5^ As a result, periodontitis is the major cause of tooth loss and edentulism in adults worldwide.^1,5,6^ Various studies have demonstrated that periodontitis is associated with several systemic diseases and conditions, including diabetes,^7^ cardiovascular diseases,^8^ and adverse pregnancy outcomes.^9^ Furthermore, periodontitis shares common risk factors with non-communicable diseases, notably lifestyle factors (stress, smoking, and alcoholism), metabolic factors (obesity), and socioeconomic factors.^4,10^ However, although periodontitis can be easily prevented or detected and treated in the majority of cases, it remains a global epidemic poorly known, poorly screened, and undertreated because the early stages of the disease are asymptomatic, with the majority of affected patients seeking care late in the disease process.^6^ Therefore periodontitis is a major public health problem due to its high prevalence and negative impacts, both on oral health (tooth loss and disability, aesthetics problems, and masticatory dysfunction) and on general health and quality of life (systemic impacts, undernutrition), resulting in significant psychosocial and economic repercussions.^11,12^ To reduce the burden of periodontitis, early and appropriate diagnosis and treatment are essential. Furthermore, the identification of the most severe cases of the disease is important, as they are the most difficult from a therapeutic point of view, presenting teeth with a worse prognosis, a greater risk of tooth loss, and a higher likelihood of influencing the systemic health of individuals.^13^ The 2018 EFP/AAP new classification of Periodontal and Peri-Implant Diseases and Conditions, based on the stages and grades of periodontitis,^4,13^ may allow better diagnosis and treatment of periodontitis. In Côte d’Ivoire, epidemiology data of periodontitis are sparse. Therefore, this study aimed to describe the prevalence and severity of periodontitis in patients attending the Periodontics Service of the Cocody University Hospital at Abidjan, in Côte d’Ivoire.

## Methods

### Study population

The subjects were recruited from patients attending the periodontics service at the Odonto-Stomatological Consultations and Treatment Center (CCTOS) of the Cocody University Hospital from 2014 to 2018. The CCTOS is an important university center in Abidjan, Côte d’Ivoire, which provides oral health services for all social classes and all regions of the country. Routinely in the periodontics service at the CCTOS, all patients answer a general and oral health questionnaire, including sociodemographic characteristics, behavior, and comorbidities, and undergo a full-mouth periodontal and radiographic examination.

The study included individuals aged ≥20 years, who had at least six teeth in the dental arch, and their files were complete. Two trained investigators (CNT and AAN) jointly analyzed the files of 2221 patients during the study period. The final sample, meeting the selection criteria, consisted of 596 patients. The anonymity of information included in clinical files was guaranteed. The research project was approved by the Scientific Committee of the Odontostomatology Training and Research Unit, University Félix Houphouët Boigny of Abidjan (approval number 2019/428).

### Periodontal clinical examination

The periodontal examination was performed using William’s manual periodontal probe (Michigan O probe, Hu-Friedy Mfg. Co., Chicago, IL, USA) at six sites per tooth (mesiobuccal, buccal, distobuccal, mesiolingual, lingual, and distolingual) by dental students, systematically trained and calibrated, under the supervision of periodontists. Third molars were not included. The following parameters were recorded: plaque index (PI) (O’Leary et al^14^),bleeding on probing (BOP) (Ainamo and Bay^15^),tooth mobility (Mühlemann^16^), missing teeth, probing depth measurement (PD), gingival recession (GR), and clinical attachment loss (CAL). PD was measured from the free gingival margin to the bottom of the pocket/sulcus. CAL was defined as the distance from the cementoenamel junction to the bottom of the pocket/sulcus.

### Diagnostic criteria for periodontitis case definition

Periodontitis was diagnosed, according to the 2018 EFP/AAP new classification of Periodontal and Peri-Implant Diseases and Conditions, as follows: a subject presenting with interdental CAL at two non-adjacent teeth or buccal/oral CAL≥3 mm with pocketing >3 mm was diagnosed with periodontitis.^13^ This classification is based on the stages and grades of periodontitis. In the present study, only stages were considered. Periodontitis severity staging was defined by the interproximal CAL at sites with the greatest attachment loss: a CAL of 1–2 mm was defined as Stage I (mild periodontitis), 3–4 mm as Stage II (moderate periodontitis) and ≥5 mm as Stages III–IV (severe periodontitis). In this study, Stage I/II were classified as ‘non-severe periodontitis’ (i.e., mild and moderate periodontitis combined: CAL<5 mm), and Stages III/ IV were considered severe periodontitis (CAL≥5 mm).

The extent of periodontitis has been characterized as localized (≤30% of the sites involved) and generalized (>30% of the sites involved) or molar/incisor pattern.

### Sociodemographic variables

The sociodemographic, behavioral, and medical parameters studied were age, gender, profession, dia­betes mellitus, smoking, alcoholism (assessed by presence or absence), oral hygiene habits (daily brushing frequency). Age was stratified in groups of 20-34, 35-49, 50-64, and 65-80 years. According to the National Institute of Statistics classification of Côte d’Ivoire,^17^ professions were grouped into three socioeconomic categories: high (middle and senior managers…), medium (civil servant agents and employees, military sub-officers…), and low (the unemployed, pupils and students, non-salaried workers such as the shopkeepers, craftsmen, laborers). Toothbrushing frequency was categorized as never, 1, 2, or ≥3 times a day.

### Data analysis

Statistical analysis was performed for all variables using SPSS 22.0 for Windows 10 (SPSS Inc., Chicago, IL, USA). Descriptive statistics were calculated: numbers, percentages, means, and standard deviations of quantitative variables, proportions of qualitative variables. Correlations of sociodemographic, behavioral, and clinical characteristics and the extent and severity of periodontitis were calculated by Pearson’s chi-squared test for the comparison of percentages, and one-factor ANOVA was performed to compare the means between two independent groups. Logistic regression analysis was performed to model the relationship between the extent and the severity of periodontitis and covariables as potential risk indicators. The odds ratio (OR) and their 95% confidence intervals (CI) were calculated. The level of statistical significance was set at P<0.05.

## Results

The sociodemographic, behavioral, and medical characteristics of the sample are presented in Table 1. Males represented 59.23% of the sample. The mean age was 44.94±14.34 years, ranging from 20 to 80 years; the 35-49 age group (35.23%) was the most represented. The majority of patients (59.06%) were in the low socioeconomic category. Regarding comorbidities, lifestyle, and oral hygiene habits, 6.04% of patients were diabetics, 4.87% were active smokers, 23.66% were alcohol users, and 63.42% brushed their teeth twice a day (Table 1).

**Table 1:** Sociodemographic, behavioral, and medical characteristics of the study population

**Characteristics**	**No.**	**%**
**Gender**		
Male	353	59.23
Female	243	40.77
**Age** (years) (no.; mean ± SD)	44.94 ± 14.34
20-34	163	27.35
35-49	210	35.23
50-64	164	27.52
65-80	59	09.90
**Socioeconomic category**		
Low	352	59.06
Medium	171	28.70
High	73	12.24
**Diabetes mellitus**		
Yes	36	06.04
No	560	93.96
**Smoking**		
Yes	29	04.87
No	567	95.13
**Alcohol intake**		
Yes	141	23.66
No	455	76.34
**Toothbrushing frequency**		
Never	03	00.50
Once/day	186	31.21
Twice/day	378	63.42
>3 times/day	29	04.87
**Total**	596	100

**Figure 1:**
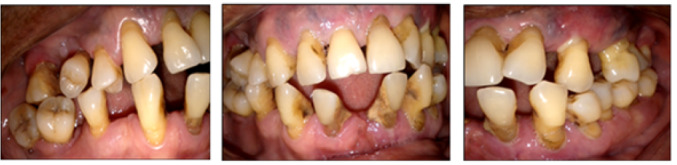
Clinical front and side views of generalized stage IV grade C periodontitis of a 65-year-old diabetic patient. Note the presence of severe attachment loss, with dental migrations/mobility and missing teeth.

**Figure 2:**
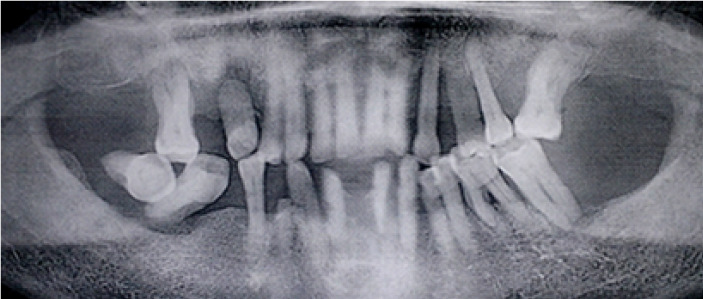
A panoramic x-ray of the patient presented in Figure 1. Note severe generalized radiographic bone loss extending to the middle or apical third of the root.

Periodontal clinical characteristics are described in Table 2. The plaque index (PI) value was 78.9±19.1%, and the mean bleeding on probing (BOP) was 57.2±21.6%. The prevalence rates of stage I/II periodontitis and stage III/IV periodontitis were 37.42% and 62.58%, respectively. The prevalence of localized periodontitis (LP) and generalized periodontitis (GP) was 60.23% and 39.77%, respectively. PD ≥6 mm was present in 47.15% of the sample. One-third (34.90%), one-quarter (25.50%), and two-thirds (64.77) of patients had gingival recession, mobile teeth, and missing teeth, respectively. Radiographically, horizontal bone lysis (33.39%) and mixed bone lysis (41.11%) were the most observed (Figures 1 and 2) (Table 2).

**Table 2:** Periodontal Clinical and radiographic characteristics distribution of the study population

**Characteristics**	**No.= 596**	**%**
**PI** (%, mean ± SD) 78.9 % ± 19.1
**BOP** (%, mean ± SD) 57.2 % ± 21.6
**Probing depth** (mm)	
4-5	315	52.85
≥ 6	281	47.15
**Gingival recession** (mm)	
Yes	208	34.90
No	388	65.10
**Clinical attachment loss** (mm)	
1-2	02	00.34
3-4	221	37.08
≥ 5	373	62.58
**Extent**		
Localized	359	60.23
Generalized	237	39.77
**Tooth mobility**		
Yes	152	25.50
No	444	74.50
**Missing teeth**	
0	210	35.23
1 à 4	229	38.42
≥ 5	157	26.35
**Bone loss**		
Vertical	152	25.50
Horizontal	199	33.39
Mixed	245	41.11

PI = plaque index, BOP = bleeding on probing

**Figure 3:**
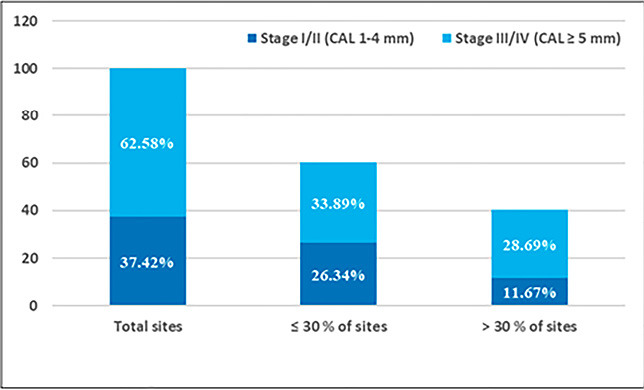
Distribution of periodontitis according to the stage and extent.

Regarding the severity of periodontitis, a higher prevalence of NSP (stage I/II) was observed in the youngest age group than in the other age groups. The frequency of NSP (stage I/II) decreased with age, from 36.32% in the 20-34 years of age to 8.97% in the 65-80 years of age; in contrast, older subjects had a higher prevalence of SP (stage III/IV) than younger subjects, except for the 65-80 age group. Patients in the low socioeconomic category had a higher prevalence of SP than the high socioeconomic category (66.48% vs. 07.23%). The SP was significantly associated with age (P<0.001) and socioeconomic status (P=0.001) (Table 3). The distribution of periodontitis according to severity and extent of NSP or SP showed that the prevalence of LP (≤30% of sites involved) in NSP and SP was 26.34% and 33.89% (including malar/incisor pattern), respectively; the prevalence of GP (>30% of sites involved) in NSP and SP was 11.07% and 28.69%, respectively (Figure 3).

**Table 3:** Distribution of periodontitis according to demographic, socioeconomic and behavioral factors

**Characteristics**	**Stage I/II** **No. (%)**	**Stage III/IV** **No. (%)**	**Total** **No. (%)**	**P-value**
**Gender**				P=0.989
Male	132 (59.19)	221 (59.24)	353 (59.23)	
Female	91 (40.81)	152 (40.76)	243 (40.77)	
**Age** (years)				P=0.000*
20-34	81 (36.32)	82 (21.98)	163 (27.35)	
35-49	78 (34.98)	132 (35.39)	210 (35.23)	
50-64	44 (19.73)	120 (32.17)	164 (27.52)	
65-80	20 (08.97)	39 (10.46)	59 (09.90)	
**Socioeconomic category**				P=0.001*
Low	104 (46.63)	248 (66.48)	352 (59.06)	
Medium	73 (32.73)	98 (26.27)	171 (28.70)	
High	46 (20.62)	27 (07.23)	73 (12.24)	
**Diabetes mellitus**				P=0.380
Yes	11 (04.93)	25 (06.70)	36 (06.04)	
No	212 (95.07)	348 (93.30)	560 (93.96)	
**Smoking**				P=0.467
Yes	9 (04.04)	20 (05.36)	29 (04.87)	
No	214 (95.96)	353 (94.64)	567 (95.13)	
**Alcohol intake**				P=0.518
Yes	56 (25.11)	85 (22.79)	141 (23.66)	
No	167 (74.89)	288 (77.21)	455 (76.34)	
**Toothbrushing frequency**				P=0.223
Never	02 (00.90)	1 (0.27)	03 (00. 50)	
Once/day	78 (34.97)	108 (28.95)	186 (31.21)	
Twice/day	134 (60.09)	244 (65.42)	378 (63.42)	
>3 times/day	09 (04.04)	20 (5.36)	29 (04.87)	
**Total**	223 (37.42)	373 (62.58)	596 (100)	

*Chi-squared test: Significant differences (P<0,05)

The periodontal indices are shown in Table 4. The mean number of missing teeth increased significantly with age, ranging from 1.28±2.40 to 5.94±5.41, with more severe tooth loss in the older subjects (65-80 years of age) than in the younger subjects (20-34 years of age) (5.94±5.41 vs. 1.28±2.40; P<0.001). Compared to non-smokers, active smokers had significantly greater mean CAL (6.60±2.45 vs. 5.98±1.99; P=0.009). Diabetics had a significantly greater mean CAL (6.25±1.91 vs. 5.99±2.03; P<0.001) than non-diabetics. Age, diabetes, and smoking were significantly associated with the SP (Table 4).

**Table 4:** Periodontal clinical characteristics (mean ± SD) according to gender, age, smoking, and diabetes

**Characteristics**	**Missing teeth**	**GR**	**PD**	**CAL**
**Gender**	2.90 ± 3.96	1.14 ± 1.77	4.87 ± 0.94	5.99 ± 2.00
Male				
Female	3.52 ± 4.35	1.11 ± 1.76	5.00 ± 0.88	6.03 ± 2.05
**Age** (years)				
20-34	1.28 ± 2.40*	0.66 ± 1.39	4.80 ± 0.74	5.45 ± 1.69
35-49	3.10 ± 3.85*	1.06 ± 1.75	4.92 ± 0.84	5.90 ± 1.97
50-64	4.08 ± 4.52*	1.48 ± 1.82	5.09 ± 1.06	6.56 ± 2.09
65-80	5.94 ± 5.41*	1.68 ± 2.26	4.73 ± 1.21	6.36 ± 2.47
**Smoking habit**				
Non-smoker	3.12 ± 4.14	1.10 ± 1.73*	4.91 ± 0.93	5.98 ± 1.99*
Smoker	3.76 ± 4.01	1.63 ± 2.32*	5.04 ± 0.62	6.60 ± 2.45*
**Diabetes**				
Non-diabetic	3.10 ± 4.11	1.12 ± 1.77	4.91 ± 0.93*	5.99 ± 2.03*
Diabetic	4.06 ± 4.43	1.21 ± 1.70	5.03 ± 0.69*	6.25 ± 1.91*
**Total**	3.15 ± 4.13	1.13 ± 1.76	4.92 ± 0.92	6.01 ± 2.02

SD: standard deviation; probing depth; GR: gingival recession; CAL: clinical attachment loss. *One-factor ANOVA: Significant differences (P<0.05).

Table 5 presents the logistic regression analysis of the association between the outcome variable, SP (stage III/IV), and GP (>30% of sites affected) and sociodemographic, behavioral, and clinical predictor variables. Older age group (OR=1.59, 95% CI: 1.11-2.26, P=0.010), having ≥5 missing teeth (OR=2.31, 95% CI: 1.08-4.89, P=0.029), alcohol intake (OR=2.61, 95% CI: 1.12-6.05, P=0.026), and poor oral hygiene habits (OR=2.22, 95% CI: 1.22-4.08, P=0.009) were identified as independent risk indicators for SP. Subjects in the age group (≥35 years) and those with ≥5 missing teeth had a higher risk of having GP, compared to individuals in the younger age group and those who had <5 missing teeth, respectively. There was a significantly higher risk for SP in alcohol users compared to non-users and in subjects who had a low frequency of toothbrushing (<2 times/day) compared to those who had routine toothbrushing (≥2 times/day) (Table 5).

**Table 5:** Logistic regression for factors associated with SP and GP as the dependent variables

**Variable**	**GP (N=237)**		**Stage III/IV (N=373)**	
	**OR (95% CI)**	**P-value**	**OR (95% CI)**	**P-value**
**Age** (years)				
20-34	1	0.010*	1	0.170
≥35	1.59 (1.11-2.26)		1.30 (0.89-1.90)	
**Alcohol intake**				
No	1	0.081	1	0.026*
Yes	2.18 (0.90-5.25)		2.61 (1.12-6.05)	
**Toothbrushing frequency**				
≥2 times/day	1	0.650	1	0.009*
<2 times/day	0.88 (0.51-1.51)		2.22 (1.22-4.06)	
**Missing teeth**				
<5	1	0.029*	1	0.852
≥5	2.31 (1.08-4.89)		0.92 (0.42-2.02)	

GP: generalized periodontitis; Stage III/IV periodontitis; OR: odds ratio; CI: 95% confidence interval

## Discussion

Using a full-mouth periodontal examination, this retrospective study assessed the prevalence and severity of periodontitis and associated risk indicators in adult subjects 20-80 years of age, who attended the Odonto-Stomatological Consultations and Treatments Center of the Cocody University Hospital at Abidjan. The prevalence of stage I/II periodontitis was 37.42%, and the prevalence of stage III/IV periodontitis was 62.58%.

Periodontitis was mostly localized (60.23%) compared to generalized (39.77%). Periodontitis is highly frequent among adults worldwide.^18-23^ The prevalence of severe periodontitis in the present study was higher than in several other studies in adults: 46.68% in France,^18^ 40.2% in South Africa,^23^ and 15.4% in Portugal.^22^ According to the EFP-AAP classification for clinical practice,^4,13,24^ the frequency of subjects with stages III/IV periodontitis was 54% in Turkey^25^ and more than 30% in China.^26^ In west Africa, the prevalence of subjects with generalized stage IV grade chronic periodontitis was 50.4% in Senegal.^27^ However, the lack of standardization of diagnostic criteria for periodontitis limits the comparison between studies, depending on different case definitions.

The mean age of the sample was 44.94±14.34 years. Periodontitis was more common in men (59.20%) than in women. Similar observations were made in France (58% men vs. 42% women),^18^ in the United States (56.4% men vs. 38.4% women),^19^ and Senegal (63.8% men vs. 37.2% women).^27^ Periodontitis was significantly more frequent in the low socioeconomic category than in the high socioeconomic category (59.10% vs. 12.24%; P=0.006). The risk of periodontitis follows a socioeconomic gradient, with subjects with the lowest income and education levels being at the highest risk.^28^ The socioeconomic level of a given population is a good marker for several risk factors of periodontal diseases, such as oral hygiene, access to oral care, and behaviors.^29^ Lack of health insurance and lack of financial resources were barriers to access to oral health care; therefore, this category of patients was referred to periodontists at CCTOS, a public university dental hospital, with affordable rates. However, consultations were often late because of the insidious nature of periodontitis, the low awareness of periodontal health in the general public, and the lack of periodontal disease management by general practitioners in Côte d’Ivoire. As a result, aesthetic and functional problems (gingival recession, spontaneous loss of teeth, tooth hypermobility with pain, and masticatory dysfunction) were common, needing complex periodontal care and rehabilitation.

Furthermore, regarding oral hygiene, the majority of patients had poor oral hygiene (mean PI=78.9±19.1%) and moderate to severe gingival inflammation and bleeding (mean BOP=57.2±21.6%). Lack of regular toothbrushing increased the risk of severe periodontitis (OR=2.22, 95% CI: 1.22-4.08) significantly. The level of oral hygiene and the presence of periodontal inflammation is an essential clinical parameter concerning the assessment of periodontitis treatment outcomes and the residual disease risk after treatment.^13^ In addition to local risk factors, periodontitis was related to behavioral risk factors such as alcohol intake and smoking; and general risk factors such as diabetes. Smoking and diabetes are strong risk factors associated with severe periodontitis.^4,7^ In fact, in this study, diabetics had more generalized and more severe periodontitis than non-diabetics. In a previous study in Abidjan, severe periodontitis was associated with poor diabetes control.^30^ The effective control of these risk factors requires integrated management of the patients.^10^

All these factors resulted in a high periodontitis burden in the study population, with severe tissue destruction. One-third of the patients (34.90%) had at least one gingival recession area. These mucogingival defects had a significant esthetic impact, which is a frequent reason for consultation. The mean pocket depth was 4.92±0.92 mm. PD≥6 mm was noted in 47.15% of patients. A higher prevalence of PD≥6 mm (50.4%) was found in Senegal.^27^ The mean CAL was 6.01±2.02 mm, and 62.30% were severe with CAL≥5 mm (stage III/IV periodontitis). This severe tissue destruction resulted in significant rates of tooth mobility (25.50%) and high rates of missing teeth (64.90%). The average number of missing teeth per subject was 3.15±4.13, with extremes of 1 to 25 teeth lost for periodontal reasons. These high rates of tooth loss in our sample were similar to those reported in Senegal, with 5±4 teeth lost (0 to 17 teeth).^27^ Apart from spontaneous tooth loss due to late consultations, tooth extraction remains the most common treatment in low-income countries due to the lack of adequate technical facilities and the low socioeconomic level of the populations.^31^ In addition, most often, the edentulous condition was not restored,^32^ resulting in masticatory deficits that could lead to undernutrition, impacting the quality of life and general health of patients.^11,12,31,33^ Radiographically, bone lysis was horizontal in 74.53% of subjects. In generalized forms, the proportion of horizontal bone lysis was the highest (57.89%), whereas, in localized forms, angular lysis was the most frequent (20.63%). The observed differences were statistically significant (P=0.000).

The high prevalence of severe periodontitis associated with a high rate of edentulism highlights the lack of prevention and effective interventions to reduce the burden of periodontal disease in Côte d’Ivoire. Periodontitis is a public health concern as a chronic inflammatory disease that shares risk factors and social determinants with other high-mortality non-communicable diseases.^12^ Measures are needed to provide access to oral health care and institute periodontal health promotion policies, in line with WHO strategies for NCDs through the common risk factor approach.^10^

The present study had some limitations. The prevalence of periodontitis could be underestimated due to the retrospective manual collection of data and the number of incomplete files not included in the sample. The clinical examinations were performed by several students and their supervisors, which might have led to differences between examiners despite the calibrations. As it was a hospital-based study limited to the Abidjan district, caution is warranted when generalizing the results to the Ivorian populations.

## Conclusions

This study revealed a high prevalence of severe periodontitis (stage III/IV) and a high rate of edentulism, with severe aesthetic and functional consequences. The severity of periodontitis was associated with age, socioeconomic status, diabetes, and smoking. Delayed time to management was related to a lack of financial resources. Awareness and screening actions, through appropriate public health programs, to provide access to periodontal care, would allow early diagnosis and management of periodontitis and its risk factors, according to a common approach to risk factors for non-communicable diseases, to improve the health and well-being of our populations. These results will serve as a basis for future population-based cross-sectional epidemiological studies with larger samples.

## Authors’ contributions

KCN and MG contributed to conception and design. PZ contributed to data acquisition and analysis. KCN, PZ, and MG contributed to data interpretation and drafted and critically revised the manuscript. All authors gave their final approval and agreed to be accountable for all aspects of the work

## Availability of data

The datasets used and/or analyzed during the current study are available from the corresponding author on reasonable request.

## Ethics approval

The research project was approved by the Scientific Committee of the Odontostomatology Training and Research Unit, University Félix Houphouët Boigny of Abidjan (approval number: 2019/428).

## Competing interests

The authors declare no conflict of interest related to the study.
